# Hospital Meetings

**Published:** 1907-03-09

**Authors:** 


### HOSPITAL MEETINGS.

### MIDDLESEX HOSPITAL.

The annual meeting was held on February 28, Mr. Henry Morris, President of the Royal College of Surgeons and consulting surgeon to the hospital, in the chair.

### The Year's Report.

The report for the year showed a gratifying increase in subscriptions and donations, as well as a noticeable reduction in expenditure. The year began, however, with a deficit of nearly £7,500, which has been entirely wiped off by the conspicuous generosity of Mr. H. L. Bischoffsheim, who presented £5,000, and subsequently joined with Mrs. Bischoffsheim in a donation of £2,500. By this means the debt on the chapel has been completely wiped off. In grateful acknowledgment of this timely assistance, the Board decided to name a ward "Bischoffsheim Ward."

During the year 4,018 in-patients and 46,771 out-patients were treated. The figures for 1905 would supply an unsatisfactory basis of comparison, owing to the unavoidable closing of the building for repairs for part of the time. The electrical and light department gave satisfactory results.

### The Year's Losses and Gains.

Lord Cheylesmore, Chairman of the Board, in moving the adoption of the report, alluded with deep regret to the loss the hospital had sustained in the death of Mr. Edward Ashby Pardon, who for twenty-eight years held the position of resident medical officer. He had never ceased to take a personal interest in the hospital, and his work had conduced largely to its improved efficiency and usefulness. His Lordship also expressed regret at the resignation through ill-health of Sir Arthur Townley Watson, Bart., K.C., Deputy-Chairman of the weekly Board, Chairman of the Council of the Medical School, and the Board's representative on the Central Hospital Council for London. Great satisfaction was felt by all concerned with the arrangement now being made for acquiring, in conjunction with the medical school of St. Mary's Hospital, the lease of a sports ground. It was hoped that this would prove of incalculable benefit to their own medical school.

### Cancer Research.

According to the report of the Cancer Charity, the average number of beds occupied daily was the same as that for the last two years—namely, 44.5. Amongst the donations received was one of £1,050 from Mrs. Arthur Busk to endow a bed in the cancer wing in memory of her late husband, and another of similar amount as the joint gift of Mr. F. Coston Taylor and his sister, Mrs. J. W. Western, to endow a bed in Greenhow Ward. Dr. A. Lazarus Barlow has been reappointed director of the Cancer Research Laboratories. The appointment was also renewed of Dr. Victor Bonney to the Walter Emden Research Scholarship, and of Dr. Louis Courtauld to the Richard Hollins Scholarship.

### THE CANCER HOSPITAL, FULHAM ROAD.

The fifty-sixth annual meeting was held on Wednesday, February 27, Lord Ludlow presiding. From the report of the Committee it appeared that during the year 1906 there were 817 new in-patients, as against 713 in the previous year, while the daily average number of beds occupied was 99, an increase of 11. In the out-patient department 1,698 new cases were treated, the total number of attendances for the year being 17,376. Owing to the deficiency of the ordinary income, it has been found necessary to realise between £6,000 and £7,000 from capital to meet expenses. The charity receives no grants from the Hospital Saturday or Sunday Funds or the King's Fund. In September last a deputation from the medical staff was sent to the International Congress for the Study of Cancer held in Heidelberg and Frankfurt-on-Main, and the opportunity was also taken by the delegates to visit hospitals and scientific institutions in Germany, Denmark, and the Netherlands, where they gathered much valuable information. During the year there has been a considerable increase in the Cancer Research and Pathological Department, and further laboratory accommodation and a large museum have been added; the latter will permit of many valuable pathological and microscopical specimens being exhibited to the best advantage. Lord Ludlow, in opening the meeting, referred in terms of sympathy and regret to the great loss the hospital had sustained by the death of its Chairman, Lieut.-Col. Parr. The vacant chair had been unanimously offered to Mr. Owen E. Hayter, and his acceptance was regarded with much satisfaction and pleasure. Owing to increase of work and number of patients the nursing staff had to be considerably augmented. Only last year Lady Ludlow opened the new nursing home, but that did not afford sufficient accommodation, and eventually it would be necessary to turn their attention to enlarging the home.

The reports and balance-sheet were adopted, and on passing the usual votes of thanks special mention was made of the hospital's indebtedness to the metropolitan and provincial Press for their continued advocacy of the charity.

